# Spectrophotometric assays for evaluation of Reactive Oxygen Species (ROS) in serum: general concepts and applications in dogs and humans

**DOI:** 10.1186/s12917-021-02924-8

**Published:** 2021-06-26

**Authors:** Camila Peres Rubio, José Joaquin Cerón

**Affiliations:** Interdisciplinary Laboratory of Clinical Analysis (Interlab-UMU), Veterinary School, Campus of Excellence Mare Nostrum, University of Murcia, Campus de Espinardo s/n, 30100 Murcia, Spain

**Keywords:** Biomarkers, Free radicals, Oxidants, Oxidative stress, Peroxides, Total oxidant status

## Abstract

Reactive oxygen species (ROS) are reactive compounds derived from oxygen. In biological systems, an excessive amount of ROS can cause oxidative damage to biological macromolecules being involved in different diseases. Several assays have been developed in the last 30 years for ROS evaluation. The objective of this article will be to provide an update about the spectrophotometric methods currently used in the assessment of ROS in serum. The chemical basis of four different techniques will be reviewed, and examples of their possible applications will be provided. A particular emphasis about the practical applications of these assays in the dog will be made, but selected information about their use in humans will also be presented for comparative purposes, following a One-Health approach. The information about the spectrophotometric assays presented in this paper should be interpreted with caution once limited information about them is available yet, and further studies should be performed to clarify what they measure and their clinical application. Ideally, when applied to evaluate a sample’s oxidative status, they should be incorporated in a panel of analytes where other oxidants, antioxidants, and biomarkers of inflammation were also included.

**Supplementary Information:**

The online version contains supplementary material available at 10.1186/s12917-021-02924-8.

## Background

Oxidants and antioxidants are produced by living organisms in their metabolic activity. The balance between the two is tightly regulated, and it is essential for maintaining cellular and biochemical functions. An unbalance between oxidant production and antioxidants in favour of the former, leading to cellular signalling disruption and chain reactions, is defined as oxidative stress [1].

Oxidants are compounds generated endogenously as a result of aerobic metabolism in physiological conditions [1]. They can have a physiological role since, during inflammation, they are produced by neutrophils and macrophages for the destruction of pathogens; however, if the redox homeostasis is disrupted and oxidants are produced at too high levels, they can produce tissue damage and contribute to disseminating the inflammation [2–4].

Antioxidants are natural or synthetic molecules that protect a biological target against oxidative damage. They act by preventing the uncontrolled production of oxidants, intercepting their reactions with biological structures, and repairing the damage caused by oxidative stress. They can be endogenously synthesised, which can be enzymes such as superoxide dismutase, catalase, and the glutathione peroxidase/glutathione reductase system, or non-enzymatic compounds such as peroxiredoxins, ceruloplasmin, ferritin, and albumin. But also there are exogenous or diet-derived antioxidants such as tocopherols, carotenes, ascorbate, and some minerals (e.g., Zn, Mn, Se). Exogenous antioxidants act synergistically with the endogenous ones; however, it has been described that endogenous defences are more protective [2, 5–9].

If the antioxidant system can not counterbalance an excessive production of oxidants, these may indiscriminately target and produce damage to proteins, lipids, polysaccharides, and DNA [2, 10]. These oxidant compounds produced include those derived from the oxygen, called reactive oxygen species (ROS) and those derived from other molecules different from oxygen: reactive nitrogen species (RNS) as nitric oxide and nitric peroxide, reactive carbon species (RCS), and reactive sulphur species (RSS) [11]. This review will focus on ROS compounds, the biomarkers currently most frequently used for evaluating the oxidant status in both animals and humans.

### Concept of ROS

**Fig. 1:**
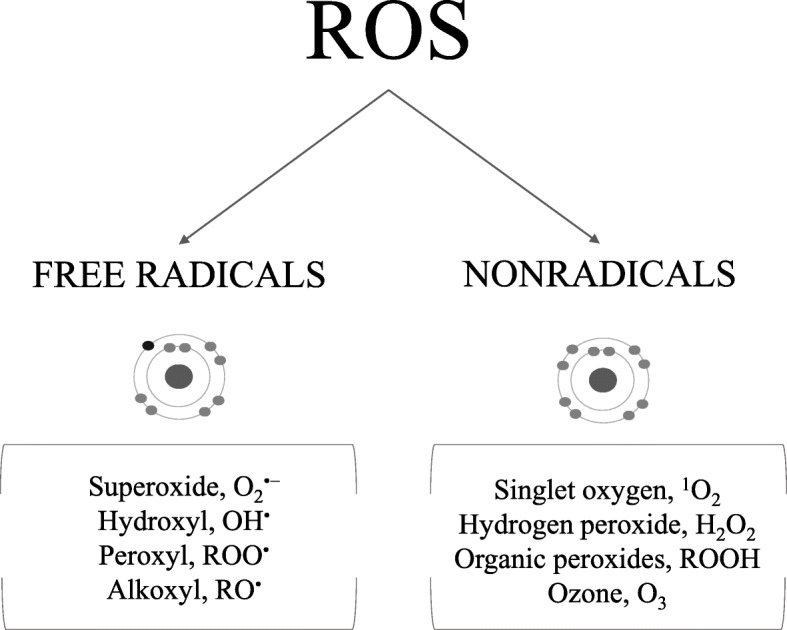
Reactive oxygen species (ROS) of major interest in oxidative stress

ROS is a collective term used to describe oxygen-derived small and reactive molecules. Those include free radicals (molecules containing one or more free electrons), such as superoxide (O_2_^•−^), hydroxyl (OH^•^), peroxyl (ROO^•^), and alkoxyl (RO^•^), and nonradicals molecules (with paired electrons) such as singlet oxygen (^1^O_2_), hydrogen peroxide (H_2_O_2_), organic peroxides (ROOH, hydroperoxides), and ozone (O_3_), among others (Fig. 1) [4, 12–14]. These nonradicals molecules can produce oxidation “per se” or can also be converted into free radicals.

The most important source of ROS in cells is probably the mitochondrial electron-transport chain, but they can also be generated in different cellular locations, such as the endoplasmic reticulum or nucleus. In addition, some ROS such as ROOH can also be formed after the oxidation of different compounds such as lipids, proteins or DNA [15, 16].

The biological lifetime of each ROS is different (Table 1) [17–20]. For example, although O_2_^•−^ has a half-life of seconds, ROOH derived from proteins (PrOOH), in the absence of light, heat, reducing agents, and metal ions that can degrade them [21, 22], were stable during 2 h at 37 °C in neutral aqueous solutions [23].

**Table 1:** Approximate half-lives of reactive oxygen species (ROS)

Molecule		Half-Life (at 37 °C)
Free radicals	O_2_^•−^	10^− 6^ s
	OH^•^	10^−9^ s
	ROO^•^	7 s
	RO^•^	10^−6^ s
Nonradicals	^1^O_2_	10^− 6^ s
	H_2_O_2_	chemically stable
	ROOH	In some cases, such as some PrOOH, until 2 h

Adapted from 12, 15, 18

ROS can contribute to different physiological functions, especially in the immune system, such as controlling fibroblast proliferation and differentiation or proper folding and maturation of immunoglobulins [15, 16]. However, as previously stated to oxidant compounds, ROS can become toxic and cause damage to biomolecules when their concentrations are uncontrolled, a situation associated with several diseases in animals and humans [12, 24–27]. 

### Evaluation of ROS

ROS, particularly the free radical molecules, are difficult to quantify in biological fluids due to their high reactivity [28–30]. Most of them persist for only a short time in vivo and cannot be measured directly [11]. Thus, for accurate detection and characterisation of ROS, complex techniques such as electron spin resonance, spin-trapping, or pulse radiolysis should be used [31–33]. These techniques can be labour-intensive and time-consuming, and they may also require sophisticated and expensive instrumentation, facts that limit their general use [34].

As an alternative, ROS can be estimated by the products generated during the damage that they can produce to the different biomolecules  [11, 35].Some examples of these products are F2-isoprostanes, malondialdehyde (MDA), and ROOH derived from lipids (LOOH)  as the phosphatidyl-choline hydroperoxide (PcOOH), that are compounds produced during the lipid damage; or 8-hydroxy-2′-deoxyguanosine produced in case of DNA damage. They can be measured acurately by gas or high-performance liquid chromatography (HPLC) techniques involving post-column chemiluminescence detection, reductive-mode electrochemical, or coupling to a tandem mass spectrometry, although commercially ELISA kits are also available to their estimation [36–42]. 

 In the two above-described situations, these techniques used are complex and difficult to be used in routine high throughput  analysis. Therefore, spectrophotometric assays, which are more simple and easier to set up, have been developed and used to estimate ROS. Possibly the most known assays in this group are the thiobarbituric acid reactive substances (TBARS) or advanced oxidation protein products (AOPP) that evaluate some compounds produced during lipid and protein oxidation, respectively. TBARS is considered an unspecific technique for MDA determination and can produce false increases of MDA generated by the heating step of the assay and also by the interaction with a variety of other compounds, like bile pigments, saturated and unsaturated aldehydes, sucrose, amino acids, and urea [43–49]. The AOPP assay measures oxidatively modified albumin and di-tyrosine containing cross-linked proteins [50]. Despite their limitations, both assays are still widely used because of their simplicity [11, 51].

In addition to TBARS and AOPP, other spectrophotometric assays that have not been so widely studied can also measure ROS molecules, including those produced during oxidative damage.

In this review, the focus will be on these later assays, which have been less studied and used in general. It should be noted that these spectrophotometric techniques have two main general limitations:

- They do not measure all the ROS molecules, and they are not specific to individual ROS. Therefore, they can just be used to estimate the ROS concentration in the sample [52–54].
- When applied in serum or plasma, the ROS compounds with a short half-life possibly have disappeared from the sample, and these assays probably will only measure the most stable ones, such as H_2_O_2_ and ROOH. Therefore, the spectrophotometric assays will estimate the more stable ROS in serum or plasma after blood processing.

### Objectives and aspects to cover in this review

The objective of this article will be to provide an update about the spectrophotometric techniques, different to TBARS and AOPP, that can be currently used for the assessment of ROS in serum. To the author’s knowledge, there is a published review of different spectrophotometric assays that can be used in canine serum for the measurement of total antioxidant capacity (TAC) [55]; however, there are no similar reviews about the spectrophotometric evaluation of ROS.

Overall, four different spectrophotometric methods will be presented, and each of them will be described: (1) the chemical basis, (2) their advantages and drawbacks, (3) studies and applications in dogs, and (4) selected information from the human side for comparative purposes. A particular emphasis on the dog will be given in this review; since in this species, there is evidence that different infectious, parasitic, metabolic diseases and other conditions such as stress and ageing are associated with oxidative stress [56–62]. Therefore there is a growing interest in studying oxidative stress in the dog from a clinical perspective. In addition, this species is gaining importance as an experimental model to study human diseases and biological processes related to oxidative stress [63]. Additionally, we will also provide selected information about reports in humans for comparative purposes, following a One-Health approach. It is expected that this review will be of use for researchers in bioveterinary sciences and could help to better use and interpretation of ROS measurements.

## Main text

### Total oxidant status measurement based on ferrous ion–o-dianisidine complex (TOS-dianisidine) assay

This assay, also named “total oxidant status”, measures mainly the H_2_O_2_ and LOOH [34]. In a dose-response study, the assay gave linear and appropriate responses with H_2_O_2_, *t*-butyl (*t*-Bu-OOH) and cumene ROOH (Cu-OOH) pure solutions [34]; therefore, it could measure at least these compounds in serum.

**Fig. 2:**
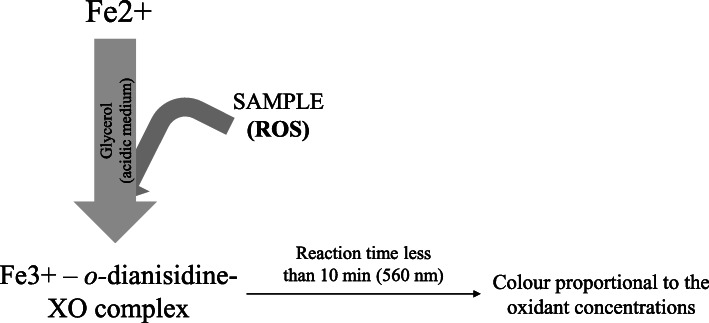
An overview of the total oxidant status measurement based on ferrous ion–o-dianisidine complex (TOS-dianisidine) reaction

The reaction’s basis consists of the oxidation of Fe^2+^ by ROS of the sample. This yields F^3+^ and OH^•^/RO^• ^in an acid reaction mixture containing ferrous sulphate and *o*-dianisidine diluted in H_2_SO_4_ . These Fe^3+^ can be detected by using the dye xylenol orange (XO; *o*-cresolsulfonphthalein-3′,3″-bis(methyliminodiacetic acid sodium salt)), which binds Fe^3+^ forming a complex that absorbs between 540 and 580 nm (Fig. 2) [34, 64, 65]. The TOS-dianisidine assay is commonly calibrated with H_2_O_2,_ and the results are expressed as μmol/L H_2_O_2_.

In this assay, the oxidation reaction rate is enhanced by using glycerol molecules. Besides, the inclusion of *o-*dianisidine allows a prolonged lifetime of reagents and the prevention of serum proteins’ precipitation during the reaction period, making the assay suitable for routine clinical analysis and easy to adapt to automated analysers [34].

TOS-dianisidine showed adequate stability when serum samples of dogs were stored at − 80 °C for a year [66]. However, it showed low stability with canine samples stored at 25 °C for 24 h, at 4 °C for 72 h and at − 20 °C for a year [66]. In human samples, the serum concentrations were not affected by storage at 4 °C for 1 day or at − 80 °C for 3 months [34].

#### Advantages and drawbacks

The TOS-dianisidine assay has some advantages [34]:

- it is quick and easy to perform,
- it is precise,
- there are commercially available kits for its measurement,
- the reagents are easy to prepare, and their lifetime is prolonged,
- it can be easily automated.

However, the assay presents some drawbacks:

- haemolysis and bilirubin interfere with the reaction,
- EDTA inhibited the colour formation,
- *o*-dianisidine is a carcinogenic and toxic substance.

#### Studies in dogs

The results from studies that determined serum TOS-dianisidine in dogs are shown in Table 2. This table shows that TOS values  could differ depending on the surgical procedure [67–69] and that they decreased after anaesthesia [68, 70]. TOS-dianisidine was increased in dogs with sarcoptic mange, canine monocytic ehrlichiosis, leishmaniosis and anaemia compared to healthy dogs [71–74]. However, no difference in this assay was observed between different clinical leishmaniosis presentations and before and after treatment against canine leishmaniosis [74, 75], and pneumoperitoneum and hyperbaric oxygen therapy did not produce significant changes [76, 77].

**Table 2:** Studies in which total oxidant status method based on ferrous ion–o-dianisidine complex (TOS-dianisidine) assay was applied in serum samples of dogs

Situation studied	Concentrations (μmol H_**2**_O_**2**_ Equiv./L)	Reference
Orthopaedic surgery for treatment of fractures or luxation of limbs	Before: ± 100.0 After: ± 170.0	[67]
Laparoscopic ovariectomy	Before: 10.9 After: 22.3	[69]
Open ovariectomy	Before: 11.5 After: 34.5	[69]
Periimplantitis with ibuprofen treatment	One-week implant: ± 70.0 8 weeks implant + placebo: ± 42.0 8 weeks implant + ibuprofen: ± 30.0	[68]
Thiopental anaesthesia and surgical trauma	Before: 12.7 After: 13.5	[70]
Propofol anaesthesia and surgical trauma	Before: 25.3 After: 19.7	[70]
Sarcoptic mange	Control: 12.2 Diseased: 20.3	[71]
Canine monocytic ehrlichiosis	Control: 6.6 Mono-infected: 17.2 Co-infected: 19.0	[72]
Leishmaniosis	Control: 6.8 Stage I: 34.0 Stage II: 32.0 Stage III: 33.0 Stage IV: 34.0	[74]
Different severity of anaemia	Control: 4.2 Mild: 7.0 Moderate: 6.8 Severe: 6.6	[73]
Leishmaniosis before and after treatment	Before: ± 8.0 30 days after treatment: ± 5.0 180 days after treatment: ± 18.0	[75]
Intra-abdominal pressure	* All pressures tested - before induction of anaesthesia: ± 7.0 * Control - only anaesthesia: ± 7.0 * 30 min and 24 h after deflation: 12 mmHg: ± 9.0 15 mmHg: ± 10.0 * 7 mmHg: ± 7.0 (in all time-points)	[76]
Effects of ovariohysterectomy	Before surgery: ± 0.8 6 h after surgery: ± 0.1 18 h after surgery: ± 0.8 30 h after surgery: ± 0.05	[77]
Effects of ovariohysterectomy and hyperbaric oxygen therapy	Before surgery: ± 0.6 6 h after surgery: ± 0.03 18 h after surgery: ± 0.04 30 h after surgery: ± 0.05	[77]
Serum pools from healthy Beagle dogs	6.4–9.1	[66]

*±*, approximately (data based on article figures)

#### Studies in humans

 TOS-dianisidine changes in human patients depending on the surgical procedure [78, 79] and anaesthesia [79]. In addition, their concentrations increased in patients suffering from major endemic zoonoses such as tuberculosis and acute brucellosis [80, 81] and decreased after bacterial meningitis treatment in children [82] (See Additional file 1: Table S1).

### Ferric-xylenol orange (FOX) assay

According to previous reports, the FOX assay could measure at least the following four ROS molecules: H_2_O_2_, linoleic ROOH (Lo-OOH), *t*-Bu-OOH and Cu-OOH [83, 84].

**Fig. 3:**
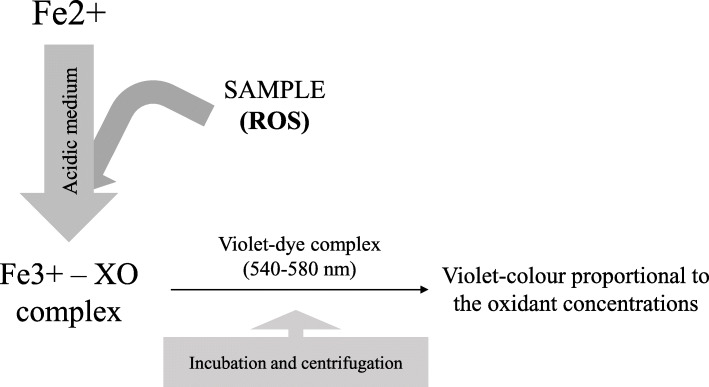
An overview of ferric-xylenol orange (FOX) reaction

The first version of the FOX assay (FOX I) was based on the oxidation of Fe^2+^ to Fe^3+^ in acidic solution by ROS compounds present in the sample and its detection by XO (Fig. 3) [64, 65, 85, 86]. The Fe^3 +^ −XO complex sign is read against known concentrations of H_2_O_2_ or *t*-Bu-OOH [85, 87]. The main differences of this assay with TOS-dianisidine are that the *o*-dianisidine and glycerol compounds are not used here, and that this assay includes incubation periods of 30 min minimum and centrifugation steps [85]. Although sorbitol has been used to stimulate the chain reaction of Fe^2+^ [84, 85], it should be pointed out that it causes extensive peroxidation of lipid in the FOX assay itself, leading to a false signal [84, 88, 89]. An automatic version of FOX I, in which protein precipitation and centrifugation step are avoided, has been validated [87]. In addition, iron D-gluconate was used instead of ferrous ammonium sulphate to improve the reagents’ stability [87].

As FOX I was not suitable for measuring low levels of LOOH, a second and improved version (FOX II) was developed, which included a butylated hydroxytoluene/methanol system. This allowed a better measurement of ROOH - including LOOH - in plasma samples [90]. The adaptation of the assay to automated analysers improved its use [26, 71, 91, 92]. FOX was stable when serum samples of dogs were stored at − 80 °C for a year [66], and in plasma of humans was stable for at least 1 month when stored at − 20 °C [87]. However, FOX results  in serum of dogs increased after storage at 25 °C for 72 h and at − 20 °C for 360 days [66].

#### Advantages and drawbacks

The FOX assay has as advantages [86, 87]:

- it can be automated,
- the FOX II allowed the measurement of LOOH.

On the other hand, the assay also presents some drawbacks [34, 86, 87]:

- the reagent used in the manual version shows a continuous darkening of the solution, making it stable only for less than 6 h,
- the assay could require a centrifugation step depending on the version,
- the ascorbic acid and other compounds that bind Fe^3+^ through competition with XO (e.g., desferrioxamine, diethylenetriaminepentaacetic acid, ethylenediaminetetraacetic acid) interfere with the reaction,
- it is influenced by haemolysis,
- blood collected on EDTA is unsuitable for analysis.

#### Studies in dogs

FOX results from previous studies can be found in Table 3. FOX was significantly higher in dogs with sarcoptic mange [71], idiopathic inflammatory bowel disease [26] and atopic dermatitis [92] when compared to healthy dogs. However, no difference was observed between dogs with canine monocytic ehrlichiosis and healthy dogs [91].

**Table 3:** Studies in which the ferric-xylenol orange (FOX) assay was applied in serum samples of dogs

Situation studied	Concentrations (μmol ***t***-Bu-OOH Equiv./L, unless stated otherwise)	Reference
Sarcoptic mange	Control: 6.9 μmol H_2_O_2_ Equiv./L Diseased: 15.5 μmol H_2_O_2_ Equiv./L	[71]
Idiopathic inflammatory bowel disease	Control: 72.0 Diseased: 148.0	[26]
Atopic dermatitis	Control: 65.0 Diseased: 82.0	[92]
Canine monocytic ehrlichiosis	Control: ± 80.0 Subclinical disease: ± 80.0 Clinical disease: ± 85.0	[91]
Serum pools from healthy Beagle dogs	76.0–94.6	[66]

*±*, approximately (data based on article figures)

#### Studies in humans

FOX was significantly increased in patients with various diseases, including idiopathic dilated cardiomyopathy, epilepsy [93, 94], end-stage renal disease [95], human immune deficiency virus (HIV) [96], hepatitis C [97] and malaria [98]. In addition, it decreased significantly after therapy against HIV [96] and malaria [98]. FOX has also been measured to evaluate the effect of different anaesthetic procedures and in patients with brucellosis and tuberculosis [79–81] (See Additional file 1: Table S2).

### Reactive oxygen metabolites derived compounds (d-ROMs) assay

**Fig. 4:**
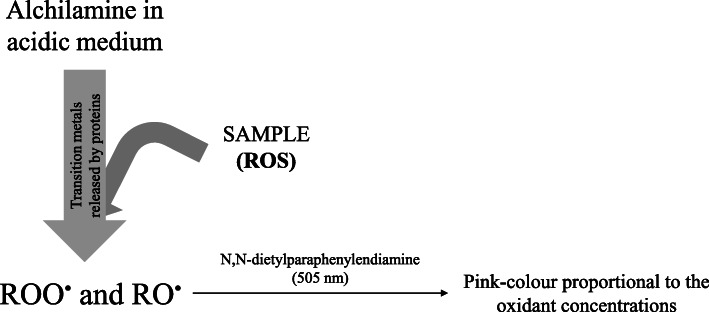
An overview of reactive oxygen metabolites derived compounds (d-ROMs) reaction

The d-ROMs assay measures the ROOH and H_2_O_2,_ although the exact ROS components that measures have not been described yet [99]. This test is based on Fenton’s reaction, which consists of indirect estimation of total ROOH in a solution test by monitoring *N,N*-dyethyl-*para*phenyldiamine radical cation (DEPPD^•−^) concentration. This radical cation originates from the diamine oxidation by ROO^•^ and RO^•^ that result from the reaction between peroxides present in the sample and the iron ions (Fe^2+^, Fe^3+^) released by the proteins in the acidic medium [100]. Such radicals are then trapped by alchilamine present in the reaction medium [100, 101]. The concentrations of these newly formed radicals (DEPPD^•−^), which have a pink colour, are measured at 505 nm, and they are directly proportional to the peroxides present in the sample (Fig. 4). The d-ROMs results are expressed in arbitrary units, the Carratelli Units (U. CARR.), which are the difference between absorbances multiplied by 10,000. It has been found that 1 U. CARR. corresponds to 0.08 mg/100 mL H_2_O_2_ [99, 100].

The concentrations of d-ROMs were stable in human serum samples when they were stored at 4 °C for 24 h and at − 80 °C for 3 months [102]. However, the validity of this assay has been questioned. Previous studies demonstrated that d-ROMs, in a dose-response study, gave no response with H_2_O_2_, *t*-Bu-OOH and Cu-ROOH pure solutions [34]. In addition, it has been shown that ceruloplasmin is a potential source of the signal detected by the test in serum from different species (mammals and birds), together with other compounds such as iron, albumin, and thiol [103, 104].

#### Advantages and drawbacks

The advantages are [99, 101, 102, 105]:

- it is simple, quick, inexpensive, and easy to set up,
- there are commercially available kits for its measurement,
- it can be adapted to automated biochemistry analysers.

Nonetheless, it also has some drawbacks [34, 100, 101, 103].

- the presence of ferroxidase enzyme (ceruloplasmin) in the sample could lead to false higher results. That is an abundant compound in serum, which could increase during inflammation,
- it is influenced by haemolysis.

#### Studies in dogs

The studies that have measured d-ROMs in serum samples of dogs are shown in Table 4. Briefly, this table shows that d-ROMs values could change depending on the oestrus cycle phase, after exercise and with antibiotic therapy after surgery [106–110]. D-ROMs decreased after antioxidant diet and increased in dogs with lymphoma and mast cell tumours [111–114]. On the other hand, differences were observed when dogs with* Leishmania* were compared with healthy dogs [115].

**Table 4:** Studies in which the reactive oxygen metabolites derived compounds (d-ROMs) assay was measured in serum samples of dogs

Situation studied	Concentrations (U. CARR)	Reference
Healthy dogs	56.4–91.4	[107]
Oestrus cycle	Proestrus: ± 85.0 Oestrus: ± 118.0 Dioestrus: ± 79.0 Anoestrus: ± 40.0	[108]
20-min aerobic hunting exercise	Before: 94.5 3 days after: 66.2	[109]
4-h aerobic hunting exercise	Before: 94.3 3 days after: 109.2	
Low-protein and fat content diet	88.0	[110]
High-protein and fat content diet	83.4	
Postsurgical antibiotic protocols - before anaesthetic induction	Control: 80.0 Amoxicillin: 83.5 BD: 94.5 SSS: 84.5 Enrofloxacin: 102.4 LS: 83.2	[106]
Postsurgical antibiotic protocols - 96 h after surgery	Control: 72.5 Amoxicillin: 77.6 BD: 71.4 SSS: 68.8 Enrofloxacin: 77.4 LS: 66.0	[106]
Long-term antioxidant-supplemented in dogs of animal assisted intervention programs	Control Before: ± 100.0 After: ± 125.0 Supplemented Before: ± 140.0 After: ± 110.0	[111]
Long-term administration of Chinese medicine	Day 0: 81.8 Last day: 68.2	[112]
Lymphoma	Control: 81.8 Diseased: 135.5	[113]
Mast cell tumour	Control: 71.0 Metastatic: 119.4 Non-metastatic: 147.5	[114]
Leishmaniosis	Control: 75.4 Patent leishmaniosis: 108.2 Clinically healthy, seropositive: 73.5 Other diseases: 127.7	[115]

*BD*, benzylpenicillin / dihydrostreptomycin; *SSS*, Sulfamethazine /sulfamerazine /sulfathiazole; *LS*, Lincomycin /spectinomycin; *±*, approximately (data based on article figures)

#### Studies in humans

Previous studies showed that d-ROMs increased in serum of patients with infections, arthritis, allergies, obesity, cancer, and metabolic disease compared with healthy subjects [101, 116] and that patients with chronic gastritis could have lower d-ROMs when ascorbic acid is supplemented [117]. Also, it was described that sex does not influence their concentrations, but age might affect them [102] (See Additional file 1: Table S3).

### Peroxide-activity (POX-act) assay

This assay measures total ROOH [101]. In addition, in previous reports, the POX-Act reacted with H_2_O_2_, *t*-Bu-OOH pure solutions [34], indicating that it should measure at least these molecules in serum or plasma samples.

**Fig. 5:**
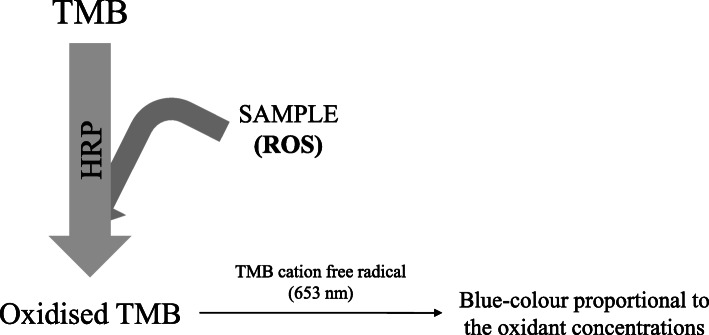
An overview of peroxide-activity (POX-Act) reaction

The POX-Act test is based on the oxidation of the chromogen substrate 3,5,3′5’-tetramethylbenzidine (TMB) by the reaction produced between the horseradish peroxidase (HRP) added in the solution and some of the ROS present in the sample (Fig. 5) [118]. Blue coloured products corresponding to the TMB cation free radical (absorbance maximum at 653 nm) are generated [118]. Results are calculated from the standard linear curve using known H_2_O_2_ concentrations by subtracting the first absorbance reading from the second [101].

#### Advantages and drawbacks

The POX-Act system has some advantages [119]:

- there are commercially available kits for its measurement,
- it is easy to perform,
- the TMB oxidation products present high stability at acid pH,
- the HRP is active over a wide pH range.

On the other hand, it also shows drawbacks such as:

- incubation of 20 min is needed, which limits automation,
- there is no available information about its stability during different times and storage conditions.

#### Studies in dogs

To the author’s knowledge, there are no studies on its use in serum samples of dogs.

#### Studies in humans

POX-Act was increased in patients after coronary intervention and in those with infections, arthritis, allergies, obesity, and metabolic disease [101, 120]. In addition, oral α-tocopherol supplementation in patients during haemodialysis [121] and rosuvastatin treatment [122] could be related to decreased POX-Act concentrations (See Additional file 1: Table S4).

### Comparative studies

A few studies have compared different spectrophotometric assays in the same clinical situations. Overall, it could be pointed out that:

- No correlation among TOS-dianisidine, d-ROMs and POX-Act, was found in humans in between healthy individuals and in osteoarthritis patients [34, 101]. This fact could be due to different factors. One could be because each specific assay could measure compounds that are not measured by the other assays. Besides, different values were obtained when pure solutions of H_2_O_2_, *t*-Bu-OOH and Cu-OOH were tested by the three assays [34], which could indicate that they can have different sensitivity to detect specific compounds. In addition, the different effects that factors such as haemolysis or lipemia could have in the assays might also contribute to these divergences.
- TOS-dianisidine and FOX showed similar results when dogs with sarcoptic mange were compared with healthy dogs [71]. In the same way, both assays were significantly correlated in human studies [34]. This fact could be explained because they have a similar chemical basis.
- Dogs with ehrlichiosis did not show significant serum FOX changes when compared with healthy dogs; nevertheless, higher ROS values were found when a luminol-based chemiluminescence assay was used [91].

It is important to highlight that none of these assays can be defined as specific for ROS. In order to gain knowledge about these techniques, several authors recommend the use of integrated panels including various assays to increase the information about their behaviour in the diverse clinical situations [34, 101, 104, 123]. 

### Other techniques that could be potentially used for the estimation of ROS in serum samples

Chemiluminescence techniques can be used for ROS estimation. They are based on detecting a light emission generated during the oxidation reaction between a chemilumigenic compound, such as luminol, and the different ROS present in the sample [124–127].

The luminol, for example, allows the detection of both extra- and intracellular levels of different ROS such as H_2_O_2_, O_2_^•−^, and OH^•^ [128]. Although applied to serum or plasma, this technique would not detect the reactive species with short half-lives such as O_2_^•−^ and OH^•^ and could potentially estimate other more stable compounds such as H_2_O_2_. Using a luminol-based chemiluminescence assay, dogs with clinical and subclinical monocytic ehrlichiosis and idiopathic inflammatory bowel disease presented higher ROS concentrations than healthy dogs [26, 91]. Besides, it has been shown that luminol-based chemiluminescence results are stable in canine serum samples stored at 25 °C for 6 h, at 4 °C during 24 h, and for 60 days at − 20 °C and − 80 °C [66]. However, it is not clear which ROS have been measured when this technique was applied in the serum of dogs, and more studies are needed to clarify that.

### Future directions

There are some aspects that should be studied in more detail in the future and would allow better use and interpretation of these assays, such as:

- the different ROS measured by each assay,
- the clinical value of the different spectrophotometric assays to evaluate ROS in serum.

In addition, further studies comparing the different spectrophotometric assays between them and other biomarkers of oxidative stress such as antioxidants, trace elements, individual oxidants, and inflammation markers in different diseases would be recommended. It would help to gain knowledge about the interpretation of these assays in clinical situations and determine which assay or assays combinations could be more helpful in the management and treatment monitoring in selected diseases.

## Conclusions

Spectrophotometric assays can be used to estimate the more stable ROS in serum such as H_2_O_2_ and ROOH and provide information about oxidative status. Most of them can set up at the laboratories without the need for high-cost equipment or reagents and, together with data from a set of tests including other markers of oxidative stress, such oxidants and antioxidants, trace elements, and acute-phase proteins , can be potentially used as a tool to help in the identification and monitoring of oxidative stress associated with diseases.

However, these assays have technical drawbacks which should be considered when used. Also, when they are applied in serum or plasma, they can not measure all ROS that the sample initially had; since many of them, due to the high reactivity, have a short half-life and would disappear from the sample during its handling. In addition, studies to determine the different reactive species that each assay measures should be encouraged to make more appropriate their use and clinical interpretation.

## Supplementary Information

**Additional file 1.** Results of studies in which the spectrophotometric assays were applied in human serum samples. In this file, the studies in humans cited in the manuscript are described in more detail, with the subjects studied and results observed for each assay. They are in tables that are named S1 to S4 according to their order in the text.

## Data Availability

Not applicable.

## Abbreviations

^1^O_2_: singlet oxygen
AOPP: advanced oxidation protein products
Cu-OOH: cumene hydroperoxide
DEPPD^•−^: *N,N*-dyethyl-*para*phenyldiamine radical cation
d-ROMs: reactive oxygen metabolites derived compounds
EDTA: ethylenediaminetetraacetic acid
FOX: ferric-xylenol orange
H_2_O_2_: hydrogen peroxide
H_2_SO_4_: sulfuric acid
HIV: human immune deficiency virus
HPLC: high-performance liquid chromatography
HRP: horseradish peroxidase
LOOH: hydroperoxides from lipids
Lo-OOH: linoleic hydroperoxide
MDA: malondialdehyde
O_2_^•−^: superoxide radical
O_3_: ozone
OH^•^: hydroxyl radical
PcOOH: phosphatidyl-choline hydroperoxide
POX-Act: peroxide-activity
PrOOH: hydroperoxides from proteins
RCS: reactive carbon species
RNS: reactive nitrogen species
ROO^•^: peroxyl radical
RO^•^: alkoxyl radical
ROOH: organic peroxides or hydroperoxides
ROS: reactive oxygen species
RSS: reactive sulphur species
TAC: total antioxidant capacity
TBARS: thiobarbituric acid reactive species
TMB: 3,5,3′5’-tetramethylbenzidine
*t*-Bu-OOH: *t*-butyl hydroperoxide
TOS-dianisidine: total oxidant status method based on ferrous ion–o-dianisidine complex
U. CARR.: Carratelli Units
XO: xylenol orange
