# Supplementary material for: Spectrophotometric assays for evaluation of Reactive Oxygen Species (ROS) in serum: general concepts and applications in dogs and humans
Source: BMC Vet Res. 2021 Jun 26;17:226. doi: 10.1186/s12917-021-02924-8 (PMC8235564; doi:10.1186/s12917-021-02924-8)
Supplement: Supplementary file 1 — Additional file 1. Results of studies in which the spectrophotometric assays were applied in human serum samples. In this file, the studies in humans cited in the manuscript are described in more detail, with the subjects studied and results observed for each assay. They are in tables that are named S1 to S4 according to their order in the text. [file 12917_2021_2924_MOESM1_ESM.docx]

**Additional file: Results of studies in which the spectrophotometric assays were applied in human serum samples.**

Table S1 Studies in which Total Oxidant Status method based on ferrous ion–*o*-dianisidine complex (TOS-dianisidine) assay has been applied in human serum samples.

| **Situation studied** | **Concentrations (µmol H_2_O_2_ Equiv./L)** | **Reference** |
| --- | --- | --- |
| Laparoscopic cholecystectomy | Preoperative: 20.0  Perioperative: 22.1  Postoperative: 21.6 | [78] |
| Surgical procedures to treat abdominal wall hernias under general anaesthesia | Preoperative: 18.0  Perioperative: 20.0  Postoperative: 27.5 |  |
|  |  |  |
| Desflurane as an anaesthetic agent during caesarean section | Preoperative: 29.8  Postoperative: 24.9 | [79] |
| Sevoflurane as an anaesthetic agent during caesarean section | Preoperative: 28.5  Postoperative: 20.3 |  |
|  |  |  |
| Acute brucellosis | Patients: 18.0  Control: 7.9 | [80] |
|  |  |  |
| Pulmonary tuberculosis | Patients: 22.8  Control: 16.1 | [81] |
|  |  |  |
| Children with acute bacterial meningitis | Before treatment: 14.9  After treatment: 11.7  Control: 9.5 | [82] |
|  | | |

Table S2 Studies in which the Ferric-Xylenol Orange (FOX) assay has been applied in human serum samples.

| **Situation studied** | **Concentrations (µmol H_2_O_2_ Equiv./L)** | **Reference** |
| --- | --- | --- |
| Desflurane as an anaesthetic agent during caesarean section | Preoperative: 13.9  Postoperative: 12.6 | [79] |
| Sevoflurane as an anaesthetic agent during caesarean section | Preoperative: 13.5  Postoperative: 11.0 |  |
|  |  |  |
| Acute brucellosis | Patients: 9.7  Control: 6.1 | [80] |
|  |  |  |
| Pulmonary tuberculosis | Patients: 5.6  Control: 4.9 | [81] |
|  |  |  |
| Chronic epileptic children – FOX2 | Untreated group: 24.3  Valproic acid monotherapy group: 16.2  Carbamazepine monotherapy group: 27.1  Phenobarbital monotherapy group: 19.7  Control:13.9 | [94] |
| Chronic epileptic children – FOX Automatic version | Untreated group: 8.5  Valproic acid monotherapy group: 7.0  Carbamazepine monotherapy group: 8.7  Phenobarbital monotherapy group: 10.3  Control: 6.0 |  |
|  |  |  |
| End‐stage renal disease | Patients: 11.4  Control: 5.0 | [95] |
|  |  |  |
| Human immune deficiency virus (HIV) | Highly active antiretroviral therapy: 1.8  Not on highly active antiretroviral therapy: 2.9  Control: 0.6 | [96] |
|  |  |  |
| Hepatitis C virus | Patients under haemodialysis: 12.9  Patients without haemodialysis: 10.9  Control: 5.2 | [97] |
|  |  |  |
| Malaria infection | Patients on antimalaria therapy: 369.4  Patients without antimalaria therapy: 247.2  Control: 216.9 | [98] |
|  | | |

Table S3 Studies in which the Reactive Oxygen Metabolites derived compounds (d-ROMs) assay has been applied in human serum samples.

| **Situation studied** | **Concentrations (U. CARR)** | **Reference** |
| --- | --- | --- |
| Infections, arthritis, allergies, obesity, and metabolic diseases (routine health care program) | Diseased: ± 500.0  Healthy: ± 350.0 | [101] |
|  |  |  |
| Advanced stage cancer patients | Baseline: 414.4  After antioxidant treatment: 315.7  Control: 172.0 | [116] |
|  |  |  |
| Chronic gastritis – low dose of vitamin C supplementation | Baseline: 125.0  Follow-up: 127.0 | [117] |
| Chronic gastritis – high dose of vitamin C supplementation | Baseline: 126.0  Follow-up: 125.0 |  |
|  |  |  |
| Women | All subjects: 311.0  Women aged ≤ 51 years = ± 270.0  Women aged ≥ 51 years = ± 350.0 | [102] |
| Men | All subjects: 291.0  Men aged ≤ 51 years = ± 260.0  Men aged ≥ 51 years = ± 330.0 |  |
| *±*, approximately (data based on article figures). | | |

Table S4 Studies in which the Peroxide-activity (POX-Act) assay has been applied in human serum samples.

| **Situation studied** | **Concentrations (µmol/L H2O2 Equiv./L)** | **Reference** |
| --- | --- | --- |
| Coronary intervention - bare metal stents | 24 h before the intervention: 437.0  After the intervention:  24 h: 517.0  48 h: 529.0  1 month: 472.0 | [120] |
| Coronary intervention - sirolimus-eluting stents | 24 h before the intervention: 444.0  After the intervention:  24 h: 448.0  48 h: 471.0  1 month: 414.0 |  |
|  |  |  |
| Infections, arthritis, allergies, obesity, and metabolic diseases (routine health care program) | Diseased: ± 400.0  Healthy: ± 80.0 | [101] |
|  |  |  |
| Oral α-tocopherol supplementation in patients during haemodialysis with iron (III) application | Α-tocopherol supplementation: 400.0  Absence of α-tocopherol supplementation: 445.0  Haemodialysis session without iron treatment: 422.0  Control: 315.0 | [121] |
|  |  |  |
| Rosuvastatin treatment - 10 mg/day | Baseline: 112.8  12 weeks: 101.9  24 weeks: 96.2 | [122] |
| Rosuvastatin treatment - 40 mg/day | Baseline: 110.4  12 weeks: 85.1  24 weeks: 82.5 |  |
|  | | |
